# Multinomial Classification Certainty: a new uncertainty metric for multinomial outcome prediction

**DOI:** 10.1007/s13748-025-00404-w

**Published:** 2025-09-29

**Authors:** Florian van Daalen, Ralph Brecheisen, Leonard Wee, Andre Dekker, Inigo Bermejo

**Affiliations:** 1 Department of Radiation Oncology (Maastro), GROW Research Institute for Oncology and Reproduction, University Maastricht Medical Centre+, Maastricht, Netherlands; 2 Department of Health Promotion, Care and Public Health Research Institute (CAPHRI), University Maastricht Medical Centre+, Maastricht, Netherlands; 3 Data Science Institute, Hasselt University, Hasselt, Belgium

**Keywords:** Uncertainty, Image segmentation, Uncertainty measure, Border detection, Predictive certainty

## Abstract

Certainty of classifications is crucial when it comes to the practical application of machine learning models. Model performance measures such as accuracy are focused on the average performance of a model. However, when a model is used in a practical setting, such as a medical clinic, it is more important to know how certain the model is of a given prediction or classification than its average performance. Unfortunately, often models only provide a final classification label, usually of the class with the highest probability. This output, however, is not sufficiently informative of the certainty of this particular classification, especially in the presence of multiple classes: the highest probability might be only barely higher than the second highest. Even when a probability distribution is provided, there is no established metric to determine if a particular classification is more certain than a different one. In this article we propose a novel metric we have termed Multinomial Classification Certainty, to represent the certainty of model predictions. We discuss why existing methods cannot represent this type of certainty and we show the mathematical meaning behind important thresholds for this new measure.

## Introduction

1,2Certainty of classification is a difficult topic within machine learning. Performance measures such as accuracy are focused on the average performance of a model[1]. However, when a model is used in a practical setting, such as a clinical environment, it is important to know how certain the model is of each prediction or classification^3^. Uncertainty is often distinguished into aleatoric or epistemic uncertainty. Aleatoric uncertainty refers to uncertainty caused by inherent random factors in the process and is therefore irreducible, whereas epistemic uncertainty can be reduced with additional information[1, 2]. For the purposes of this article we want to measure the predictive uncertainty[2] of a model. In other words, how certain is the model of its own predictions. Predictive uncertainty can have both aleatoric and epistemic causes. We wish to measure the level of uncertainty at the time of classification in order to determine how reliable this classification is. Given this purpose, we will be looking at aleatoric uncertainty.

Unfortunately, models commonly only provide a final classification label, usually based on the label that has the maximum probability[3]. This says nothing about the certainty of this particular classification; it may be the case that the maximum probability is only barely higher than the second-best option.

However, even when the set of probabilities for every possible label is given, it is difficult to determine if one classification is more certain than others, i.e. what the uncertainties of the predicted class probabilities are. This is especially relevant in a multinomial classification scenario, in other words when there are more than two classes. For example, when presented with the following two probability distributions $\{0.51, 0.49, 0.0, 0.0\}$ and $\{0.51, 0.48, 0.05, 0.05\}$ there is no established way to determine if one of these predictions is more certain than the other. If one simply bases certainty of the maximum probability then these two scenarios are equivalent. However, an argument can be made that the second scenario is more certain about the maximum probability, as the difference with the second most likely probability is bigger. This can be useful information in scenarios where in addition to knowing the most likely label, we care about how much more likely this label is than the other options. It should be noted that in this article we do not consider the uncertainty around the estimated probabilities.

In this article, we propose a novel method to measure the certainty of the majority class classifications as made by a particular model when predicting or classifying a new sample. We dub this type of certainty Multinomial Classification Certainty (*MCC*). The ability to distinguish between different multinomial classification scenarios where the most likely label is equally probable, but the probabilities of the other labels differ, makes *MCC* a useful tool. To do this we first define the type of certainty we are attempting to measure. We will then review the current approaches and discuss how these are not fit for our purposes. Subsequently, we will discuss our proposed measure. Finally, we will discuss the potential uses.

### A measure of certainty

In this article, we are specifically interested in how certain a model is of its predictions when the predictions are based on the most likely class label. We are not interested in the correctness of the classification itself. It is entirely possible that the model is very certain but also wrong.

In short, a model gives the following probability distribution $\{p(y_1), p(y_2), p(y_3), ..., p(y_n)\}$ for a given individual *x*, for all n possible labels, which result in the classification $\hat{y}$, where $\hat{y}$ is the label with the highest probability. We want to know how certain the model is that $\hat{y}$ corresponds to the true label $y^{\dagger }$ of individual *x*. This is often referred to as predictive uncertainty[2]. We are not interested in what the underlying cause of the uncertainty is; we simply want to measure it.

If the estimated probability distribution for the outcome is uniform, the model cannot do better than provide a random guess when classifying *x*. In this case, it would be completely uncertain about its classification, as no label appears more likely than any other option to the model. On the other end of the spectrum, if one probability $p(y_k) = 1$ then the model is absolutely certain of its prediction as no other label is allowed according to the given probability distribution. In addition to these two extremes, there are several other scenarios of interest which we can already rank intuitively.

For example, a scenario $\{p(y_1), p(y_2), p(y_3)\}$ where $p(y_1)=p(y_2 )=0.5$, and $p(y_3)=0$, is better than a pure uniform distribution, as at the very least $p(y_3)$ is no longer relevant according to the model. However, the model is still randomly guessing between $y_1$ and $y_2$ as they are equally likely, which means there is still a high degree of uncertainty left in this distribution. In contrast a distribution like $\{0.5, 0.49, 0.01\}$ could be considered better. While in both scenarios the likelihood of the most probable label being the true label is equal, the second scenario describes a situation where the difference with the second most probable label is bigger. This means that in this scenario the model is more certain of the most probable label relative to how certain it is about the other options. Concretely this means that in the second scenario the model can distinguish between $y_1$ and $y_2$ whereas in the first scenario it cannot distinguish between those two.

**Fig. 1:**
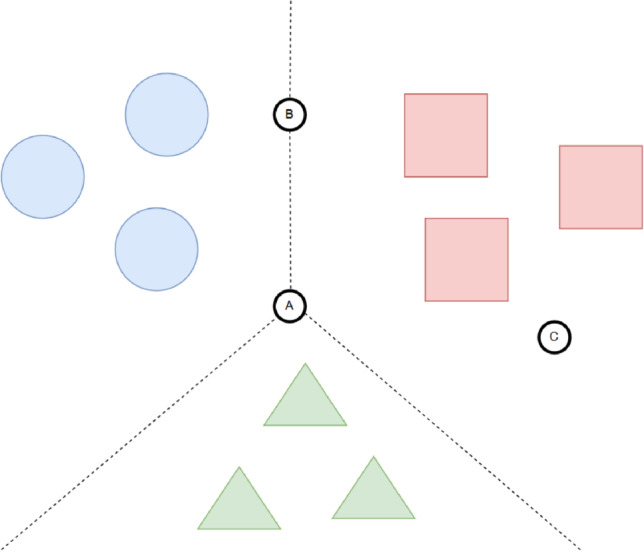
An illustrative example containing individuals belonging to 3 classes: squares triangles and circles. The class borders are indicated using dotted lines. Three new individuals (A, B, and C) that need to be classified are represented by white circles. The classification of individual A is likely to be highly uncertain, as it is on the border of all three labels. The classification of individual B should be more certain, because while it is still in the border region of two labels, it is far removed from the feature space occupied by the last label. The classification of label C should have a low uncertainty as it is solidly in the feature space occupied by one label

To illustrate the different scenarios, we have included figure 1. Figure 1 shows 3 classes: squares, triangles, and circles. It also shows the borders between these classes according to some model *M*. There are three individuals *A*, *B*, *C* which need to be classified. A falls on the border between all three classes, as such the probability distribution for *A* will be $\{0.33, 0.33, 0.33\}$ ($\{p(square), p(triangle), p(circle)\}$) as the model cannot distinguish between any of the labels for individual *A*. This would be a classification with high uncertainty. Individual *B* falls on the border between 2 classes but is definitely not a part of the triangle class, as such its probability distribution will look somewhat like this $\{0.49, 0.02, 0.49\}$. This classification for *B* is more certain as it has successfully eliminated 1 potential label, but it still has a high uncertainty as it cannot decide between the remaining two labels. Individual *C* is solidly in the area classified as squares, as such it will have a probability that looks somewhat as follows $\{0.95, 0.049, 0.001\}$. The model is very certain of the classification for this model.

In a medical context, this could be viewed as patients and their similarity to groups of patients in the training data. If a given patient is very similar to a group of patients in the training data with a certain outcome then the model will be quite certain of its prediction.

In summary, what we wish to measure can be quantified as the distance between this individual and a perfect example, in other words the Platonic ideal, of the majority class according to the current model, or conversely the distance between this individual and all of the class-borders. The measure we propose needs to be able to distinguish between these scenarios. To the best of our knowledge, there is no certainty measure that can represent this concept, which, as mentioned before, we propose to name Multinomial Classification Certainty.

Being able to distinguish between the given scenarios is important. It can be used to determine the quality of specific predictions. Currently, machine learning models are evaluated by measuring such things as accuracy and AUC. These measures give an indication of the average performance of a model, but they provide no information about the certainty of any given classification. They are simply average measures of performance. The certainty of individual classifications is an often-neglected aspect.

Individual certainty could be used to give an indication of the quality of a single classification. Based on this quality decisions could be made. For example, a classification with low certainty might warrant a second opinion from a human expert. Additionally, a high uncertainty indicates this model considers this individual to be close to a border between classes, which is in itself useful information in certain scenarios. For example, automatic image segmentation of biomedical images could use this measure to improve the detection of the borders between two types of tissue is.

## Current approaches

In this section, we will go over the various existing ways of measuring certainty and will discuss how these measures are unfit for the type of certainty we want to measure.

### AUC, Accuracy, Agreement, and Additional measures of Performance

As shortly mentioned before there are many existing methods to determine the performance of a model[4, 5], such as accuracy, AUC or F-scores. An intuitive way to represent the predictive certainty of a given classification would be to look at the model as a whole. If a model has a high accuracy, it should have high predictive certainty.

However, each of these measures share a crucial issue. All of these measures are focused on the general performance of the model. It is only an average performance, independent of how difficult it is to classify this particular individual. This makes these measures unfit to say anything about the certainty of specific individual classifications. As such, these measures can be dismissed for our purposes.

### Confidence intervals & set-prediction

Certain models can provide a confidence interval[6, 7], or provide a set prediction[8] or multinomial prediction, or provide a set prediction under ontic uncertainty [9], as opposed to providing a single value as its prediction. This approach allows a user to estimate certainty by looking at how big the confidence interval, or predicted set of values, is.

An example of this approach would be conformal prediction (CP) [10]. This is an online approach to tweak models where one sets a significance level of $\alpha$ and the resulting model will output predictions of sets, or confidence intervals for continuous predictions, which contains the true label in at least $1-\alpha \%$ of the cases.

This approach more explicitly tries to grapple with the concept of certainty and incorporates it in the training of the model. However, it still has two major problems.

First, not all models can inherently give confidence intervals or set-predictions, limiting the use of such approaches. Additionally they might not always be practically viable. For example, CP relies on a continuous feedback loop where earlier predictions are validated. It may not be realistically possible to precisely validate previous predictions, for example when validating previous predictions is costly, difficult or time-consuming. Lastly, while this does allow a user to rank predictions based on certainty (a smaller confidence interval or smaller set is better), it does not say anything about where within this confidence interval the true value lies. This still leaves the user with a significant level of uncertainty, especially when the confidence interval includes thresholds that decisions rely on. For example, let us say we have a model which is predicting some label *y* and predicts $y \in [0.4, 0.6]$ for a given record. Given this prediction $y = 0.4$, $y=0.5$, and $y=0.6$ are all equally likely. This becomes especially problematic if there is a decision boundary present in the given interval, such as the cut-off point for a medical intervention. For example, if $y>0.5$ is the decision boundary in this particular case, then this prediction is wholly uninformative as model cannot tell on which side of the decision boundary this particular individual falls.

### Monte Carlo simulation

An alternative way to measure uncertainty is to use Monte Carlo simulation[11]. This approach relies on repeatedly sampling your data, based on some known distribution, and creating an average classification based on these samples.

This approach allows one to create an average prediction with standard deviations for any underlying model. However, it is dependent on the probability distributions already being known. This means that this approach is only as accurate as the available probability distributions, which are not always known and might need to be approximated.

### Dirichlet beliefs

Dirichlet beliefs have been used to measure uncertainty of beliefs [12]. While this approach allows uncertainty to propagate through a network of connected nodes, it does require a starting point where the level of uncertainty is known. As such, this approach is limited to scenarios where the classification is based on some combination of beliefs with known uncertainties. Since these uncertainties are not generally known in our scenario, this approach is insufficient to represent *MCC*.

### Outlier detection and classification with reject option

Another potential alternative to measure uncertainty is to work with outlier detection[13], or a combination of outlier detection and decision boundary detection [14]. This approach tries to separate “outliers” from the “normal” data points. For example, by using one-class support vector machines[15]. Alternatively, it may use some distance function to determine how similar the individual that is to be classified is to the training data that was used. The resulting similarity score can then be incorporated into the prediction in some capacity. For example, a model can provide as its output both a probability of the predicted label, as well as a measure of how familiar it is with the individual that has been classified. If the individual is in a part of the feature space the model is unfamiliar with the prediction would be considered less certain.

In extremis this can even be used for classification with reject option[16]. This is an approach where a model can refuse to classify individuals that it is not sufficiently familiar with. For example, one may refuse to classify models that come from a low-density region[17], in other words, an individual for which the model has not seen many comparable training data points. The downside of this approach is that outlier detection is completely dependent on the quality of your training data. Unlike *MCC* which is independent of your data quality.

Additionally, this approach will view all under-represented groups as outliers. This may be problematic as datasets used in science are historically biased towards certain population groups[18]. Such biases would be magnified by outlier detection, as these individuals will end up being outliers by definition, whereas the aim of *MCC* is to not fall into this same trap.

### Shannon Entropy

Another potential measure for certainty is the Shannon Entropy[19]. This is a measure commonly used to measure how “pure” a set is. It uses the following equation: $-\sum _{x \in X} p(x) * log(p(x))$.

Shannon entropy can be used to measure the shape of a probability distribution, indicating how non-uniform it is. This can be used to measure how much of the set represented by this probability distribution consists of purely the majority class. Which can be viewed as a way of measuring of how certain the model is that this individual represents the majority class.

At first glance this seems perfect, the formula can easily be used to rank scenarios, is applicable for every model that outputs a probability distribution, and can be applied to individual classifications as it does not depend on overall model performance. This theoretically allows entropy to be used as a measure of uncertainty, where a high entropy corresponds to a low certainty for the prediction.

However, Shannon Entropy has one particular attribute that makes it unfit. Namely, Shannon Entropy prefers to reduce minority labels to 0 over maximizing the majority label. For example, Shannon Entropy views $\{0.5, 0.5, 0.0, 0.0\}$ as more favorable than $\{0.5, 0.49, 0.005, 0.005\}$. This happens because the former has two probabilities that have gone to zero, and as such no longer contribute to the entropy, whereas the latter still contains all 4 possible labels. However, in the former distribution it is impossible to distinguish between the two most likely classes, whereas in the latter there is a difference between the two most likely labels classes. This means Shannon Entropy will not accurately represent the certainty of the most likely label.

### Margin Confidence and Ratio Confidence

The margin confidence is a straightforward measure of certainty, which looks at the difference between the highest and second highest probability [20, 21]. This measure is very simple to implement and intuitive to understand. However, it faces a number of problems. It only looks at the absolute difference between the two top probabilities. Consequently, it will deem $\{0.5,0.49,0.01,0.0\}$ and $\{0,26,0.25,0.25,0.24\}$ to have an equivalent certainty. However, the second scenario contains a higher level of uncertainty as the relative difference between the top two probabilities is smaller. Additionally, in the second scenario all four probabilities are relatively speaking close together, whereas in the first scenario only the top two probabilities are. Which is not captured by the margin confidence. Consequently, the margin confidence fails to accurately represent the certainty of the most likely label.

The ratio confidence[20, 21] is a straightforward alternative where instead of the absolute difference the ratio between the two probabilities is taken. This allows the ratio confidence to avoid the problems the margin confidence has, but creates a problem of its own. It cannot distinguish between scenarios where the ratio between the top two probabilities is the same, but the ratio with other probabilities has changed. For example, $\{0.5, 0.5, 0.0\}$ and $\{0.25,0.24,0.25\}$ will have the same ratio confidence. However, the second probability distribution clearly contains a higher level of uncertainty as the most probable label has become less likely. While the ratio confidence can be considered an improvement over the margin confidence, and comes closest to what we are looking for, it still fails to fully grasp the certainty in the way we want.

## Multinomial Classification Certainty

Having established that the existing measures are not a good fit for the type of certainty we want to measure, we will now define our novel measure in the following subsections.

### Uncertainty

The ratio confidence will function as our starting point. However, where the ratio confidence only looks at two probabilities at any given time we will include the probabilities of all labels. This will ensure that we look at the effects of every probability in our probability distribution and not only at the effects of the two most likely labels. To take into account every label, we can simply take the sum of all the ratio confidence between every label and the most likely label. First let $\hat{y}$ be the label for which $p(\hat{y}) = max(p(Y))$, where *Y* is the full set of labels this will function as our base-uncertainty and is shown in equation 1.

1 $$\begin{aligned} U_{base} = \sum _{y \in Y} \frac{p(y)}{p(\hat{y})} = \frac{1}{p(\hat{y})}, \text {where Y is the set of labels}. \end{aligned}$$

This measure also avoids the problems encountered by Shannon Entropy, as it will focus on maximizing the gap between most likely label and the rest of labels, whereas Shannon Entropy focuses on minimizing the spread over all labels. However, $U_{base}$ still has trouble distinguishing between certain scenarios. For example $\{0.4, 0.3, 0.3, 0.0\}$ and $\{0.4, 0.2, 0.2, 0.2\}$ will have the same uncertainty value.

To improve our base function so it can distinguish between the scenarios we will utilize the inverse of the ratio confidence to represent the dominance[22] of the most likely label over the second most likely label. Label $\hat{y}$ is said to completely dominate label *y* if $p(y) \ll p(\hat{y})$, as such the ratio confidence can be used to represent the level of dominance. By including the degree to which the most likely label dominates the second most likely label it will be possible to distinguish between scenarios where the probability of the most likely label is the same, but the degree of dominance over the second most likely label differs. First let $\beta$ be the label for which $p(\beta )=max(p(Y \setminus \hat{y}))$. This can be incorporated into our base-uncertainty to create the function shown in equation 2.

2 $$\begin{aligned} U_{improved} = \left( \sum _{y \in Y} \frac{p(y)}{p(\hat{y}) \times \frac{p(\hat{y})}{p(\beta )}} \right) = \frac{1}{p(\hat{y})^{2}/p(\beta )} = \frac{p(\beta )}{p(\hat{y})^{2}} \end{aligned}$$

To improve the readability of the results we will normalize the function so that the uncertainty values fall within the range [0, 1], the normalized function is shown in equation 3.

3 $$\begin{aligned} U_{normalized} = \frac{p(\beta )}{p(\hat{y})^{2}} / n, \end{aligned}$$

Where n is the number of possible labels.

An uncertainty $U_{normalized}$ of 0 indicates perfect certainty, and it will only happen when $p(\hat{y}) = 1$. An uncertainty $U_{normalized}$ of 1 would indicate that every label is equally likely.

$U_{normalized}$ is very similar to ratio confidence, however because we incorporated the theoretical effects of every probability from the start, and not just the effects of the two most likely probabilities, the end result avoids the problems the ratio confidence has. These effects are represented by the normalization factor, as well as the summation over all possible probabilities, which can largely be simplified out of the equation as shown in equation 2.

### Multinomial Classification Certainty

Having created our uncertainty function we can now translate this into the measure of certainty presented in equation 4 for the most likely label $\hat{y}$.

4 $$\begin{aligned} \begin{aligned}&p(\beta )=max(p(Y \setminus \hat{y})) \\&MCC(\hat{y})= 1 - U_{Normalized}(\hat{y}) = 1 -\frac{p(\beta )}{p(\hat{y})^{2}}/n, \\&\text { If } p(\hat{y})=0, \text { then } MCC(\hat{y}) = -\infty \end{aligned} \end{aligned}$$

This can further be generalized to the following for any given label *y*:

5 $$\begin{aligned} \begin{aligned}&p(\beta )=max(p(Y \setminus y)) \\&MCC(y)= 1 - U_{Normalized}(y) = 1 -\frac{p(\beta )}{p(y)^{2}}/n, \\&\text { If } p(y)=0, \text { then } MCC(y) = -\infty \end{aligned} \end{aligned}$$

This tells us how certain the model is of a given label *x* based on the probability distribution it outputs as its classification. *MCC* will fall in the range $[-\infty ; 1]$ with the following two important thresholds. $MCC(y) = 0$ indicates all labels are equally probable, and thus equally certain. $MCC(y) = 1$ indicates the model is absolutely certain of this label *y*. The proves for these two thresholds can be found in lemmas 1 and 2.

#### Lemma 1

When $MCC(\hat{y}) = 0$ then all probabilities are equal.

#### Proof

If $MCC(\hat{y}) = 0$, then the following holds:

1. $$1-\frac{p(\beta )}{p(\hat{y})^{2}}/n= 0$$
2. $$\frac{p(\beta )}{p(\hat{y})^{2}}/n=1$$
3. $$\frac{p(\beta )}{p(\hat{y})^{2}}= n$$
4. $$1= \frac{p(\hat{y}^{2})}{p(\beta )}* n$$
5. By definition $$1 \ge p(\hat{y}) \ge p(\beta ) \ge 0$$
6. If $1 \ge p(\hat{y})> p(\beta ) \ge 0$ then $\frac{p(\hat{y})^{2}}{p(\beta )} >1/ n$ which implies $\frac{p(\hat{y}^{2})}{p(\beta )} * n > 1$ this would mean the equation in step 3 does not hold. Thus $p(\hat{y})$ must be equal to $p(\beta )$
7. If $$p(\hat{y})=p(\beta )$$ then $$\frac{\hat{y}^{2}}{\beta } = p(\hat{y}) =1/n$$
8. $$MCC (\hat{y}) = 0 \rightarrow p(\hat{y}) = p(\beta ) = 1/n \rightarrow \forall y \in Y; p(y)=1/n$$

$$\square $$

#### Lemma 2

When $MCC(\hat{y}) = 1$, then $p(\hat{y}) = 1$ and all other labels have probability 0.

#### Proof

If $MCC(\hat{y}) = 1$ then the following holds:

1. $$1-\frac{p(\beta )}{p(\hat{y})^{2}}/n=1$$
2. $\frac{p(\beta )}{p(\hat{y})^{2}}/n=0$, since $1 \ge p(\hat{y}) \ge p(\beta ) \ge 0$, and $n \ge 1$, and *n* is finite, this can only occur when $\lim {p(\beta ) \rightarrow 0}$
3. when $$\lim {p(\beta ) \rightarrow 0}$$ then $$\lim {p(\hat{y}) \rightarrow 1}$$
4. $$MCC (\hat{y}) = 1 \rightarrow p(\hat{y}) = 1, p(\beta ) = 0 \rightarrow \forall y \in (Y \setminus \hat{y}); p(y)=0$$

$$\square $$

This also provides us with the following two additional relevant value ranges for MCC:

- $MCC (y) < 0$; this label is significantly less probable, and thus less certain than some other labels. As such, it should be dismissed.
- $MCC (y) \in (0;1]$; this label is significantly more probable, and thus more certain than some other labels

It should be noted that this means that *MCC* is unbounded below. Due to this lack of a lower bound it is difficult to directly compare two labels $y_{1}$ and $y_{2}$ when $MCC(y_{1}) < 0$ and $MCC(y_{2}) < 0$. However, in practice 0 can be treated as a soft lower bound. When *MCC*(*y*) falls below 0 the certainty of *y* is so low that it can be dismissed out of hand in favor of an alternative that has a higher certainty.

While in most scenarios we are only interested in $MCC(\hat{y})$, where $\hat{y}$ is the most probably label, there are certain scenarios, such as when utilizing it to make set-value predictions, where we are also interested in the *MCC* of other labels. These two ranges are especially relevant in such scenarios. We illustrate how this can be used in section 3.4 for set value predictions.

Having shown the mathematical background of *MCC* we will discuss some practical examples in the next sections.

**Table 1:** Class probabilities and *MCC* for various examples where the probability distribution is improper, that is to say the sum of probabilities does not add up to 1. These examples illustrate that lemmas 1 and 2 still hold. However, when the sum of probabilities is far higher, or lower, than 1 strange behavior can be observed

Individual	Label x	Label y	Label z	*MCC*(*y*)	*MCC*(*x*)	*MCC*(*z*)
Example 1	0,400	0,400	0,400	0,000	0,000	0,000
Example 2	0,020	0,490	0,490	-407,333	0,320	0,320
Example 3	0,500	0,490	0,200	0,223	0,174	-3,859
Example 4	0,980	0,980	0,990	-0,014	-0,014	0,017
Example 5	0,049	0,950	1,000	-276,523	0,262	0,967
Example 6	0,000	0,200	1,000	$-\infty$	-9,000	0,920
Example 7	1,000	1,000	1,000	0,000	0,000	0,000
Example 8	0,150	0,300	0,200	-1,889	0,519	-0,625
Example 8	0,100	0,100	0,100	0,000	0,000	0,000
Example 8	0,100	0,200	0,000	-1,000	0,833	$-\infty$

### MCC and improperly formed probability distributions

So far *MCC* has been created under the assumption that the probability distribution is properly formed and all probabilities combined sum to 1. In this section we will briefly discuss what happens when this is not the case.

Recall equation 2 this equation included the sum over all probabilities, which can be simplified to 1. If we assume that the probability distribution is improperly formed this can no longer be simplified. This would result in the following new equation:

6 $$\begin{aligned} U_{improper}&= \left( \sum _{y \in Y} \frac{p(y)}{p(\hat{y}) \times \frac{p(\hat{y})}{p(\beta )}} \right) \nonumber \\  &= \frac{1}{p(\hat{y})^{2}/p(\beta )} = \sum _{y \in Y} \frac{p(y) \dot{p}(\beta )}{p(\hat{y})^{2}} \end{aligned}$$

This would result in the following equation for *MCC* when used with an improper probability distribution:

7 $$\begin{aligned} \begin{aligned}&p(\beta )=max(p(Y \setminus \hat{y})) \\&MCC(\hat{y})= 1 -\frac{\sum _{y \in Y} \frac{p(y) \dot{p}(\beta )}{p(\hat{y})^{2}}}{n}, \\&\text { If } p(\hat{y})=0, \text { then } MCC(\hat{y}) = -\infty \end{aligned} \end{aligned}$$

This can further be generalized to the following for any given label *z*:

8 $$\begin{aligned} \begin{aligned}&p(\beta )=max(p(Y \setminus z)) \\&MCC(z)= 1 -\frac{\sum _{y \in Y} \frac{p(y) \dot{p}(\beta )}{p(z)^{2}}}{n}, \\&\text { If } p(z)=0, \text { then } MCC(z) = -\infty \end{aligned} \end{aligned}$$

When using *MCC* with improperly formed probability distributions lemmas 1 and 2 still hold. This does mean that *MCC* can still be used. However, it does introduce unexpected behavior in certain edge-cases. Table 1 illustrates this. Going into more detail regarding these edge-cases is beyond the scope of this paper. For the remainder of this paper we will assume all probability distributions are properly formed.

### Illustrative examples

**Fig. 2:**
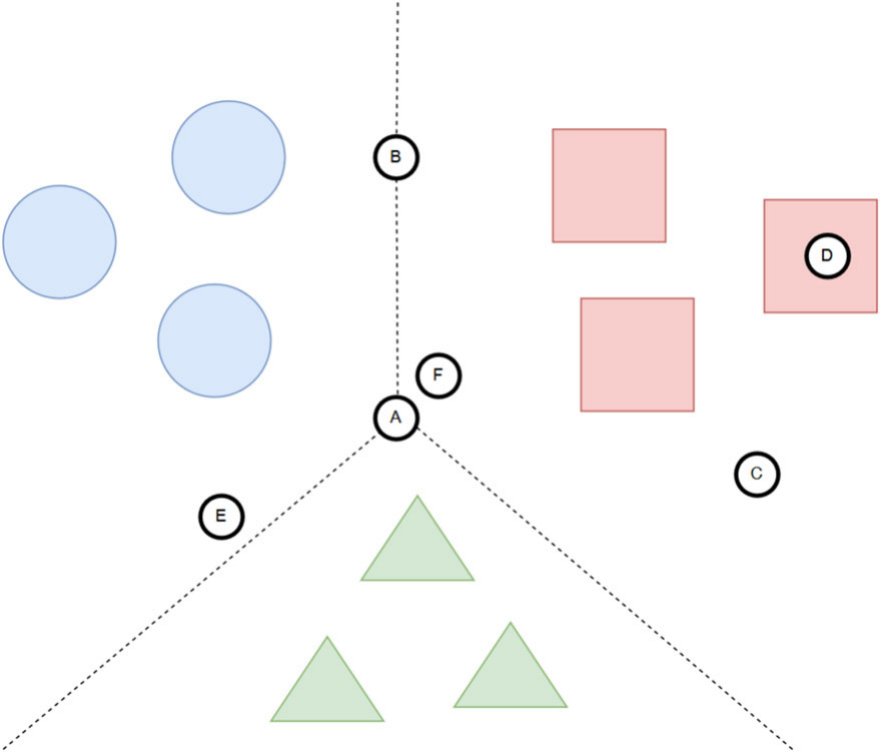
Illustrative example containing 3 classes, squares triangles and circles. The class borders are indicated using dotted lines. Several new individuals that need to be classified are indicated. Table 2 provides the class probabilities and *MCC* for each example, as well as set-values prediction based on these scores

In table 2, examples can be found of various toy scenarios, which have been classified. For each of these examples the *MCC* for each possible label is given. The examples in table 2 correspond to the examples in Fig. 2. These examples are to illustrate how *MCC* would behave in a real scenario.

**Table 2:** Class probabilities and *MCC* for the individuals in the illustrative examples shown in figure 2. The final column shows the set prediction based on the *MCC* values, using $MCC(p(y)) \ge 0$ as the inclusion criteria in the set.

Individual	Triangle	Circle	Square	*MCC*(*T*)	*MCC*(*C*)	*MCC*(*S*)	set-values prediction
A	0,333	0,333	0,333	0	0	0	$\{Triangle, Circle, Square\}$
B	0,02	0,49	0,49	-407,333	0,32	0,32	$\{Circle, Square\}$
C	0,049	0,001	0,95	-130,889	-316665,667	0,982	$\{Square\}$
D	0	0	1	$-\infty$	$-\infty$	1	$\{Square\}$
E	0,45	0,5	0,05	0,177	0,4	-65,667	$\{Triangle, Circle\}$
F	0,317	0,33	0,35	-0,108	0,05	0,093	$\{Square\}$

It should be noted that *MCC* is a stable metric when looking at the most probable label $\hat{y}$, but displays less stable behavior when looking at a less probably label $y \in (Y \setminus \hat{y})$. This is due to the fact that relatively small changes in the probability distribution can have a relatively large impact on the certainty of these less likely labels.

As mentioned, the set-value predictions in table 2 are created using using $MCC(p(y)) \ge 0$ as inclusion criteria. This behaves similarly to the naive criteria $p(y \ge 1/n$. This is expected based on lemma 1. However, it does not behave the exact same as illustrated by individual *F*. To further illustrate this we show a different set of scenarios in table 3. Here we show the *MCC* values for scenarios with various numbers of labels where $p{\hat{y}}=1/n + 0,02$ and $p(\beta )=1/n + 0,01$, as well as $p(z) = 1/n$. Despite the relatively small difference in the probabilities of each label the *MCC* scores show a meaningful difference that could be utilized. This effect becomes stronger as the numbers of potential labels grows, with *p*(*z*) progressively becoming a less certain prediction when compared to $p(\hat{y})$, despite the absolute difference between their probabilities not changing. This is especially useful when the most likely label remains relatively improbable. In this scenario *MCC* can aid a user to determine if $p(\hat{y})$ is sufficiently large compared to the other probabilities that the other labels can be dismissed.

Table 4 further illustrates how even relatively small probabilities remain relevant as the number of labels grows. In this table $p(\hat{y})$ is fixed to a value of 0, 5, additionally $p(\beta )$ is set in such a way that $MCC(\beta )=0,5$, which can be argued represents a scenario where $\beta$ should be considered a label with high certainty. In the scenario with four parties this results in $p(\hat{y}) = p(\beta )$ and $MCC(\hat{y}) = MCC(\beta )$, however, in the scenario with a hundred parties this results in $p(\hat{y}) = 5 \dot{p}(\beta )$ and $\hat{y}$ is nearly twice as certain as $\beta$, in fact $\hat{y}$ is starting to approach the limit of maximum certainty. However, $\beta$ remains relevant.

Lastly, table 5 illustrates how in scenarios with larger number of potential labels *MCC* assigns the same certainty to lower values probabilities of $p(\hat{y})$. This happens because while the probability of $p(\hat{y})$ is lower, the model is certain that $\hat{y}$ is the correct choice over a larger amount of competing labels.

**Table 3:** MCC scores for the three labels $\hat{y}$, $\beta$ *z* in scenarios with various numbers of labels, where $p(\hat{y})=1/n+0,2$, $p(\beta )=1/n+0,01$, and $p(z) = 1/n$. *z* is deemed progressively more uncertain as the number of labels grows.

Number of labels	$\boldsymbol{p(\hat{y})}$	$\boldsymbol{p(\beta )}$	$\mathbf {p(z)}$	$\boldsymbol{MCC(\hat{y})}$	$\boldsymbol{MCC(\beta )}$	$\mathbf {MCC(z)}$
4	0,270	0,260	0,25	0,108	0,001	-0,080
5	0,220	0,210	0,2	0,132	0,002	-0,100
6	0,187	0,177	0,166666667	0,155	0,003	-0,120
7	0,163	0,153	0,142857143	0,177	0,004	-0,140
8	0,145	0,135	0,125	0,197	0,005	-0,160

**Table 4:** MCC scores for the labels $\hat{y}$ and $\beta$ in scenarios with various numbers of labels, where $p(\hat{y})=0,5$, $p(\beta )$ is fixed in such a way that $MCC(\beta )=0,5$. $\hat{y}$ becomes progressively more certain as the numbers of labels grow, despite $\beta$ remaining equally certain.

Number of labels	$\boldsymbol{p(\hat{y})}$	$\boldsymbol{p(\beta )}$	$\boldsymbol{MCC(\hat{y})}$	$\boldsymbol{MCC(\beta )}$
4	0,500	0,500	0,500	0,500
5	0,500	0,447	0,642	0,500
10	0,500	0,316	0,874	0,500
20	0,500	0,224	0,955	0,500
50	0,500	0,141	0,989	0,500
100	0,500	0,100	0,996	0,500

**Table 5:** MCC scores for the labels $\hat{y}$ and $\beta$ in scenarios with various numbers of labels, where $p(\hat{y})$ and $p(\beta )$ are picked in such a way that $MCC(\hat{y})=0,9$.

Number of labels	Probability distribution	$\boldsymbol{p(\hat{y})}$	$\boldsymbol{p(\beta )}$	$\boldsymbol{MCC(\hat{y})}$	$\boldsymbol{MCC(\beta )}$
2	$\{0.854, 0.146\}$	0.854	0.146	0.900	0.789
3	$\{0.805, 0.195, 0\}$	0.805	0.195	0.900	0.701
4	$\{0.765, 0.235, 0, 0\}$	0.765	0.235	0.900	0.620
5	$\{0.732, 0.268, 0, 0, 0\}$	0.732	0.268	0.900	0.548

**Fig. 3:**
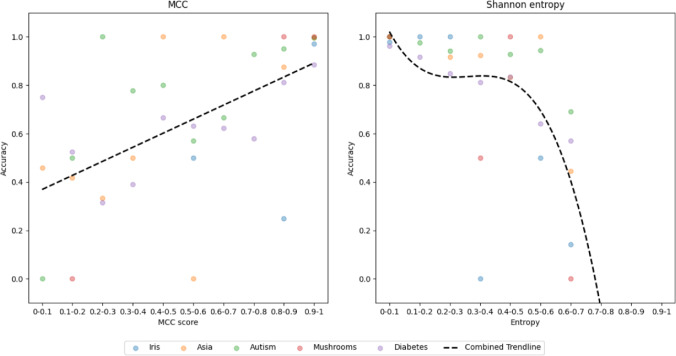
Classification accuracy plotted against *MCC* score (left) and Shannon entropy (right). *MCC* score shows a stable relation between low certainty and low accuracy. Outliers correspond to scenarios where only a handful of predictions are in a specific *MCC* range. For example, the model makes only one classification in the range $$[0.6-0.7)$$ for the Asia dataset. Shannon entropy initially shows a largely stable accuracy until the entropy increases above 0.5, at which point the accuracy suddenly plummets

**Fig. 4:**
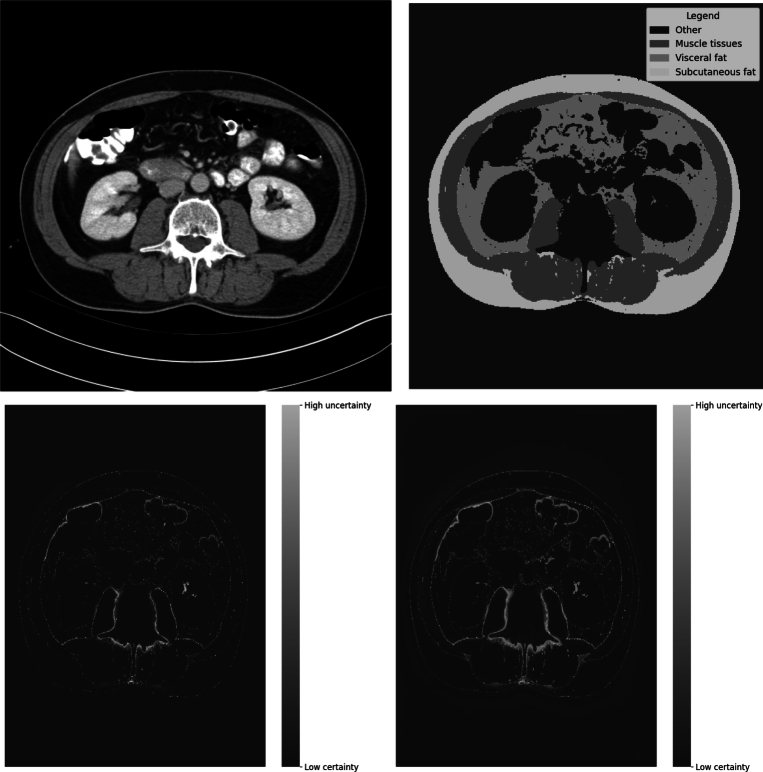
A DICOM image (top left), alongside the classifications according to max probability (top right). The bottom shows the borders as drawn based on the *MCC* for $\hat{y}$ (bottom left) and Shannon Entropy (bottom right). High levels of uncertainty correspond with the boundaries of various types of tissue according to the classification. The boundaries drawn based on Shannon entropy are less clean and show more artifacts

### Illustrating the relationship with accuracy

In this subsection the relationship between *MCC* and the accuracy of the classifications made by the model is illustrated using a number of real datasets. A comparison with Shannon entropy is shown to illustrate how *MCC* is more closely related to accuracy.

To illustrate this relationship a Bayesian network was trained on each dataset. In all cases the Bayesian network uses the K2 algorithm for structure learning, with a maximum of 5 parents per node, and the EM algorithm for parameter learning. The WEKA framework[23] was used to train the models. Performance was validated using *k*-fold cross-validation. During 10-fold cross-validation we discretized the classifications according to their *MCC* score. The following datasets where used: Iris[24], Autism[25], Asia[26], Diabetes[27],and Mushroom[28]. These datasets were chosen because they represent a variety of dataset sizes with a mix of continuous and discrete attributes. This allows us to illustrate behaviors across different datasets.

Figure 3 plots the accuracy across all datasets against the various levels of *MCC* and Shannon entropy, additionally a combined trendline is shown in each graph.

The *MCC* score shows a linear relation between certainty and accuracy. This corresponds to the desired behavior as classifications with a low certainty should represent "hard" cases, which will have a below average accuracy, whereas cases with a high certainty represent "easy" cases which have a high accuracy. Outliers correspond to scenarios where only a handful of predictions are in a specific *MCC* range. For example, the model makes only one classification in the range $$[0.6-0.7)$$ for *MCC* for the Asia dataset. As this single classification is correct it achieves a $$100\%$$ accuracy for this range.

Shannon entropy initially shows a largely stable accuracy until the entropy increases above 0.5, at which point the accuracy suddenly plummets. While "hard" cases do indeed have a higher level of entropy, the "medium" cases barely differ from the "easy" cases in terms of accuracy. It should also be noted that the Shannon entropy never rose above 0.7 for any of the classification in this experiment.

The results of this experiment indicate that *MCC* is a better indication of certainty than Shannon entropy as it is better at identifying "easy" and "hard" cases.

### Using uncertainty for image segmentation

To further illustrate the potential use of *MCC*, we now discuss a real-world use case for image segmentation. In surgical oncology, there is a trend to analyze both the quantity and quality of a patient’s muscle and fat tissue because it has been shown to be predictive of outcome, especially in certain cancer types, like pancreatic and colorectal cancer, that are associated with severe weight-loss and wasting, a process called cancer cachexia[29–32]. Muscle and fat tissue as visible on CT images at the 3rd lumbar vertebral level are commonly used to estimate body composition of these patients. Automatic methods to segment muscle and fat tissue are needed because doing it manually is too time-consuming for clinical purposes.

Figure 4 shows an example CT image at L3 level. Additionally, on the right it shows the standard segmentation result after taking assigning labels to pixels based on the maximum probability.

**Fig. 5:**
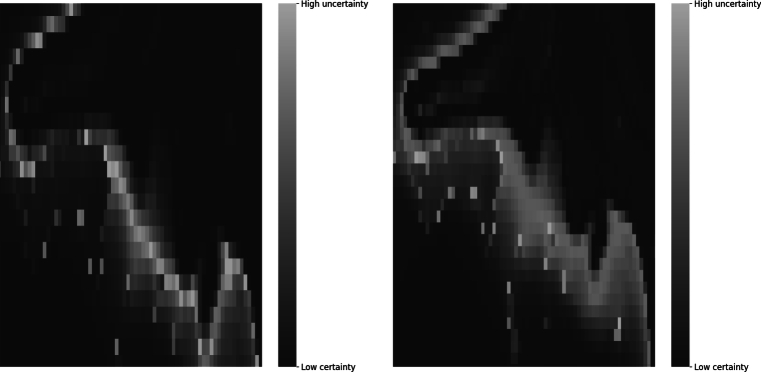
Zoomed in snapshot of figure 4 comparing borders drawn based on *MCC* (left) with borders based on Shannon entropy (right). *MCC* creates cleaner borders with fewer artifacts

**Fig. 6:**
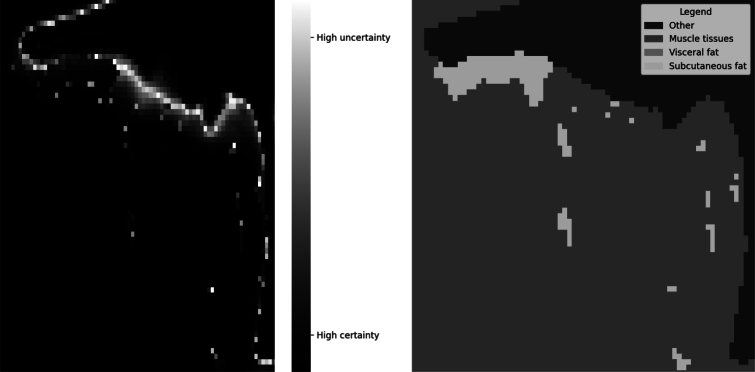
Zoomed in snapshot of the heat map & classification of figure 4. Uncertainty strongly corresponds with border area, as well as the occasional artifact represented by a single pixel of one class with no similar neighbors

Applying *MCC* to the per pixel class probabilities we can derive a certainty measure for that pixel to visualize the uncertainty of the segmentation as shown in Fig. 4. As can be seen, there are areas where uncertainty is relatively high, which mostly correspond to locations around tissue borders. Additionally Fig. 4 shows the uncertainty as based on Shannon entropy. *MCC* provides cleaner, more distinct border areas, this is especially clear when zooming in such as in Fig. 5.

This information can be used to find the borders between tissues more accurately, as intuitively the border would be wherever the uncertainty is highest. Additionally in Fig. 6 we provide a zoomed in section of the image. In this zoomed in picture, it is even more clearly visible that the areas with high uncertainty correspond to the borders between tissue, as well as some potential artifacts, such as the occasional lone pixel classified as subcutaneous fat surrounded by muscle.

## Discussion

In this article, we have proposed a new measure of classification uncertainty called *MCC* and we have shown how it works using illustrative toy examples as well as a real-life use case. While we do not go into extensive detail, we have shown that *MCC* can even be used in combination with improperly formed probability distributions. *MCC* can capture aspects of uncertainty which existing measures, such as Shannon Entropy, confidence intervals, and measures of model performance, cannot: it does not measure the overall performance of a model but the uncertainty of a single prediction; it captures the full uncertainty present within the probability distribution, regardless of its origin; it avoids the pitfalls inherent in confidence intervals; and, it does neither rely on the quality of the training dataset, (unlike outlier detection), nor on the quality of additional sources of information (such as the accurate distributions required by Monte Carlo simulation to sample from).

Its main use is to quantify the uncertainty of model predictions or classification, which can help prevent errors by rejecting predictions with high uncertainty, especially surrounding difficult to classify edge-cases. It can also be used in combination with other uncertainty quantification techniques such as Monte Carlo simulation by applying it to average output probabilities across simulations.

Furthermore, it is especially useful in situations where finding the border between two classes is valuable. This is especially relevant when using machine learning models such as support vector machines (SVMs) which rely on a decision border. Additionally, this is also relevant in scenarios where we are explicitly looking for borders of regions such as in image segmentation. Predictions in border regions inherently have high uncertainty. When this uncertainty is captured using *MCC* and used to create a heat-map, it will draw clean borders, as the borders are the large connected, but thin, regions of high uncertainty.

In fact, due to uncertainty naturally being high in border regions, both the physical border regions in image segmentation as well as the metaphorical border regions between classes in a classification task, *MCC* can explicitly be viewed as a distance function between the individual that is to be classified, and the Platonic ideal of the most likely class, according to the given model. Or conversely, as the distance function between this individual and the nearest class boundaries. Additionally, it can be used to improve confidence in machine learning models, as using *MCC* allows a model to ask human experts, or other expert systems, for help should a model not be sufficiently certain of its own classification.

While it is at its most valuable when used on the most likely label, *MCC* can also be used on the other less likely labels. Additionally, we have shown there are meaningful, objective, thresholds that can be used to compare results. This means there is no need to rely on arbitrary threshold values and allows us to avoid any problems associated with these values[33]. It should be noted that there is no hard lower bound on *MCC*. However, in practice 0 can be treated as a soft lower bound as $MCC(y) <0$ indicates that *y* has such a high level of uncertainty it is safe to dismiss *y* in favor of more certain alternatives. It should be noted that at this high level of uncertainty it also becomes meaningless to compare the *MCC* score for different labels. That is to say, both $MCC(y_{1}) = -1$ and $MCC(y_{2}) = -2$ should simply be treated as uncertain and dismissed. This effectively turns the soft-bound into a hard bound.

### Conclusion

In this article, we have proposed a new measure of uncertainty of individual classifications or predictions, which we have dubbed the Multinomial Classification Certainty (*MCC*). *MCC* captures the uncertainty present within the probability distribution given by the prediction the model produced. We have discussed the various methods currently used to quantify uncertainty, none of which methods are appropriate for multinomial classification. We have shown the various thresholds that exist and to which meaning can be attributed. This allows *MCC* to be used to identify if a given prediction should be considered relevant. We have illustrated how *MCC* behaves based on illustrative examples, various datasets, as well as a concrete image segmentation example. Additionally, we have illustrated a real-life use case in image segmentation where *MCC* can be used to easily detect the borders between different types of tissue. It is especially useful in scenarios where one is interested in finding edge-cases, the metaphorical border region between various classes, or the real, physical, border between two objects in a segmentation task.

### Future work

We would like to implement the measure in various scenarios and see how it performs in practice. This will provide a much needed empirical validation where *MCC* can be directly compared with existing measures. Unfortunately, such extensive validation and experimentation is beyond the scope of this limited paper. Currently there are plans to use it as the evaluation function in a decision tree, to use it to augment image segmentation and recognition software in a medical context, and to run experiments where noise is added to data to verify the model becomes less certain as more noise is present in the training data. Lastly, a more detailed exploration of the behavior of *MCC* regarding improperly formed probability distributions would be interesting.

## Footnotes

1 *This research received funding from the Netherlands Organization for Scientific Research (NWO): Coronary ARtery disease: Risk estimations and Interventions for prevention and EaRly detection (CARRIER): project nr. 628.011.212.*

2 *The authors have no competing interests to declare that are relevant to the content of this article.*

3 For the purposes of this article, we will be using the terms prediction and classification interchangeably
